# Extracellular Matrix Derived From Dental Pulp Stem Cells Promotes Mineralization

**DOI:** 10.3389/fbioe.2021.740712

**Published:** 2022-01-27

**Authors:** Nunthawan Nowwarote, Stephane Petit, Francois Come Ferre, Florent Dingli, Victor Laigle, Damarys Loew, Thanaphum Osathanon, Benjamin P. J. Fournier

**Affiliations:** ^1^ Dental Stem Cell Biology Research Unit, Faculty of Dentistry, Chulalongkorn University, Bangkok, Thailand; ^2^ Centre de Recherche des Cordeliers, INSERM UMRS 1138, Molecular Oral Pathophysiology, Université de Paris, Sorbonne Université, Paris, France; ^3^ Department of Oral Biology, Dental Faculty Garancière, Université de Paris, Paris, France; ^4^ Institut Curie, Centre de Recherche, Laboratoire de Spectrométrie de Masse Protéomique, PSL Research University, Paris, France; ^5^ Department of Anatomy, Faculty of Dentistry, Chulalongkorn University, Bangkok, Thailand

**Keywords:** dental pulp stem cells, gingival fibroblasts, extracellular matrix, osteogenic differentiation, decellularization

## Abstract

**Background:** Extracellular matrix (ECM) plays a pivotal role in many physiological processes. ECM macromolecules and associated factors differ according to tissues, impact cell differentiation, and tissue homeostasis. Dental pulp ECM may differ from other oral tissues and impact mineralization. Thus, the present study aimed to identify the matrisome of ECM proteins derived from human dental pulp stem cells (DPSCs) and its ability to regulate mineralization even in cells which do not respond to assaults by mineralization, the human gingival fibroblasts (GF).

**Methods:** ECM were extracted from DPSCs cultured in normal growth medium supplemented with L-ascorbic acid (N-ECM) or in osteogenic induction medium (OM-ECM). ECM decellularization (dECM) was performed using 0.5% triton X-100 in 20 mM ammonium hydroxide after 21 days. Mass spectrometry and proteomic analysis identified and quantified matrisome proteins.

**Results:** The dECM contained ECM proteins but lacked cellular components and mineralization. Interestingly, collagens (COL6A1, COL6A2, and COL6A3) and elastic fibers (FBN1, FBLN2, FN1, and HSPG2) were significantly represented in N-ECM, while annexins (ANXA1, ANXA4, ANXA5, ANXA6, ANXA7, and ANXA11) were significantly overdetected in OM-ECM. GF were reseeded on N-dECM and OM-dECM and cultured in normal or osteogenic medium. GF were able to attach and proliferate on N-dECM and OM-dECM. Both dECM enhanced mineralization of GF at day 14 compared to tissue culture plate (TCP). In addition, OM-dECM promoted higher mineralization of GF than N-dECM although cultured in growth medium.

**Conclusions:** ECM derived from DPSCs proved to be osteoinductive, and this knowledge supported cell-derived ECM can be further utilized for tissue engineering of mineralized tissues.

## 1 Introduction

Extracellular matrix (ECM) is a three-dimensional structure consisting of ground substance and fiber proteins (Theocharis et al., 2016). It provides physical support for the cells but also functions as regulatory factors in numerous biological events ranging from development to regeneration. ECM interacts with cells directly *via* cell binding domains and subsequently initiates downstream intracellular signaling (Berrier and Yamada 2007). Furthermore, ECM physical properties influence cell functions; for example, an appropriate ECM stiffness promotes adherence of specific cell population and impacts cell differentiation (Engler et al., 2006), although other external factors intervene such as topographical, geometrical, and mechanical features (Smith et al., 2018; Chu et al., 2019).

Hence, the characteristic of specific ECM and its modulations have been extensively investigated in order to control cell behaviors toward disease attenuation or healing repair. The ECM of dental pulp are rich with hyaluronan, glycosaminoglycans, and proteoglycans that are all kept together by a network of thin collagen fibrils, reticular fibrils, and fibronectin (Veis and Goldberg 2014). Due to major differences in the pulp ECM, pulp is often a non-mineralized tissue, whereas dentin is a non-collagenous ECM component. Some dentin ECM molecules function as crystal nucleators and promote crystal formation, whereas others inhibit mineralization (Goldberg and Smith 2004).

Utilization of ECM in tissue engineering has been vigorously reported. The addition of ECM to biomaterials improves their biocompatibility and enhances cell interaction (Gothard et al., 2015; Harvestine et al., 2016). The concept of decellularized ECM has been introduced as the approach for tissue engineering of complex structures (Cukierman et al., 2001). Decellularized ECM can be utilized alone or in combination with recellularization (Fu et al., 2014). Decellularized ECM contains specific signals to instruct cell response due to the distinct ECM composition and structures. To better understand this ECM, Hynes and Naba in 2012 categorized these proteins (Hynes and Naba 2012). The matrisome, the omics study of ECM proteins, had been introduced. This contains core matrisome proteins-collagens, proteoglycans, glycoproteins, and matrisome-associated proteins-regulators, secreted proteins, and affiliated proteins. Every category has been subdivided to better analyze this ECM (Naba et al., 2012).

Decellularized ECM from human dental pulp tissues supports stem cells isolated from apical papilla proliferation and differentiation *in vitro* (Song et al., 2017). The powder of decellularized dental pulp tissue enhanced angiogenesis (Bakhtiar et al., 2020). Those evidences support the benefits of oral tissue-derived ECM for regenerative approaches in dentistry. However, the limited amount of dental tissues is a major concern. *In vitro* production of ECM may circumvent this issue; stem cells combined with *in vitro* amplification could allow the production of high volume of oral stem cells-derived ECM. For example, dental pulp stem cell (DPSC)-derived ECM stimulates cell proliferation and cyclin D1 expression *in vitro* and supports dental pulp regeneration *in vivo* (Zhang et al., 2017). Dental pulp reacts to assaults through mineralization by building new dentine bridge *in vivo* or by mineralization *in vitro* (Schroder 1985; Zanini et al., 2012). This mineralization occurs through mobilization and differentiation of DPSCs (Gronthos et al., 2000).

Therefore, its ECM protein components in dental pulp are obvious research targets; we hypothesize that they are largely responsible for the physiological characteristics of this tissue. They may present an ECM signature associated with mineralization process. We performed a proteomics analysis to identify DPSC-derived ECM and to determine the potential matrisome proteins associated to mineralization. In addition, we will assess these ECM abilities to promote osteogenic differentiation in a cell which does not react to assaults by mineralization: the human gingival fibroblasts (GF).

## 2 Materials and Methods

### 2.1 Cell Isolation and Mesenchymal Stem Cell Characterization

Teeth were obtained from surgical treatment from healthy adult patients according to their treatment plan at Department of Oral and Maxillofacial Surgery, Faculty of Dentistry, Chulalongkorn University. The protocol was approved by Human Research Ethic Committee (approval number 079/2018 from Faculty of Dentistry, Chulalongkorn University). Cell isolation protocol, cell culture, and mesenchymal stem cell characterizations were performed as described in supplementary methods.

### 2.2 Extracellular Matrix Production and Decellularization

A tissue culture plate (TCP) was coated with 0.2% gelatin before cell seeding. Cells were divided into two groups. In the first group, cells were maintained in growth medium for 7 days and then changed to growth medium supplemented with 50 µg/ml of ascorbic acid for another 14 days (N-ECM group). For the second group, cells were cultured in an osteogenic induction medium, which is a growth medium supplemented with ascorbic acid 50 µg/ml, beta-glycerophosphate 5 mM, and dexamethasone 250 µM for 21 days (OM-ECM group). After 21 days, cells were decellularized by 0.5% Triton-X100 in 20 mM ammonium hydroxide and DNA removed by DNase.

### 2.3 Proteomic Analysis of Matrisome

ECM production was extracted using compartment protein extraction kit (Millipore). Proteomics analysis was performed using mass spectrometry, and the data were further processed using myProMS v3.6 (work in progress; https://github.com/bioinfo-pf-curie/myproms). Protein functions and ontology were evaluated using Metascape-A Gene Annotation & Analysis Resource website https://metascape.org/gp/index.html#/main/step1.

### 2.4 Extracellular Matrix Protein Characterization and Biological Functions

ECM production and decellularization were assessed using immunofluorescent staining of ECM proteins including type I collagen and fibronectin. Complete decellularization was confirmed by staining with phalloidin and DAPI (Guneta et al., 2017; Bombelli et al., 2018; Jeon et al., 2018; Blaudez et al., 2020). GF were reseeded on decellularized ECM. Cell proliferation was evaluated using MTT assay, and cell morphology was observed by scanning electron microscopy (SEM). For osteogenic differentiation ability, cells were maintained in osteogenic medium for 14 days; osteogenic marker gene expression was analyzed using real-time PCR (Supplementary Table S1). Mineralization ability was elucidated using alkaline phosphatase, Alizarin Red S, and Von Kossa staining.

Detailed materials and methods are found in the Supplementary Material.

## 3 Results

### 3.1 Characterizations

**FIGURE 1:**
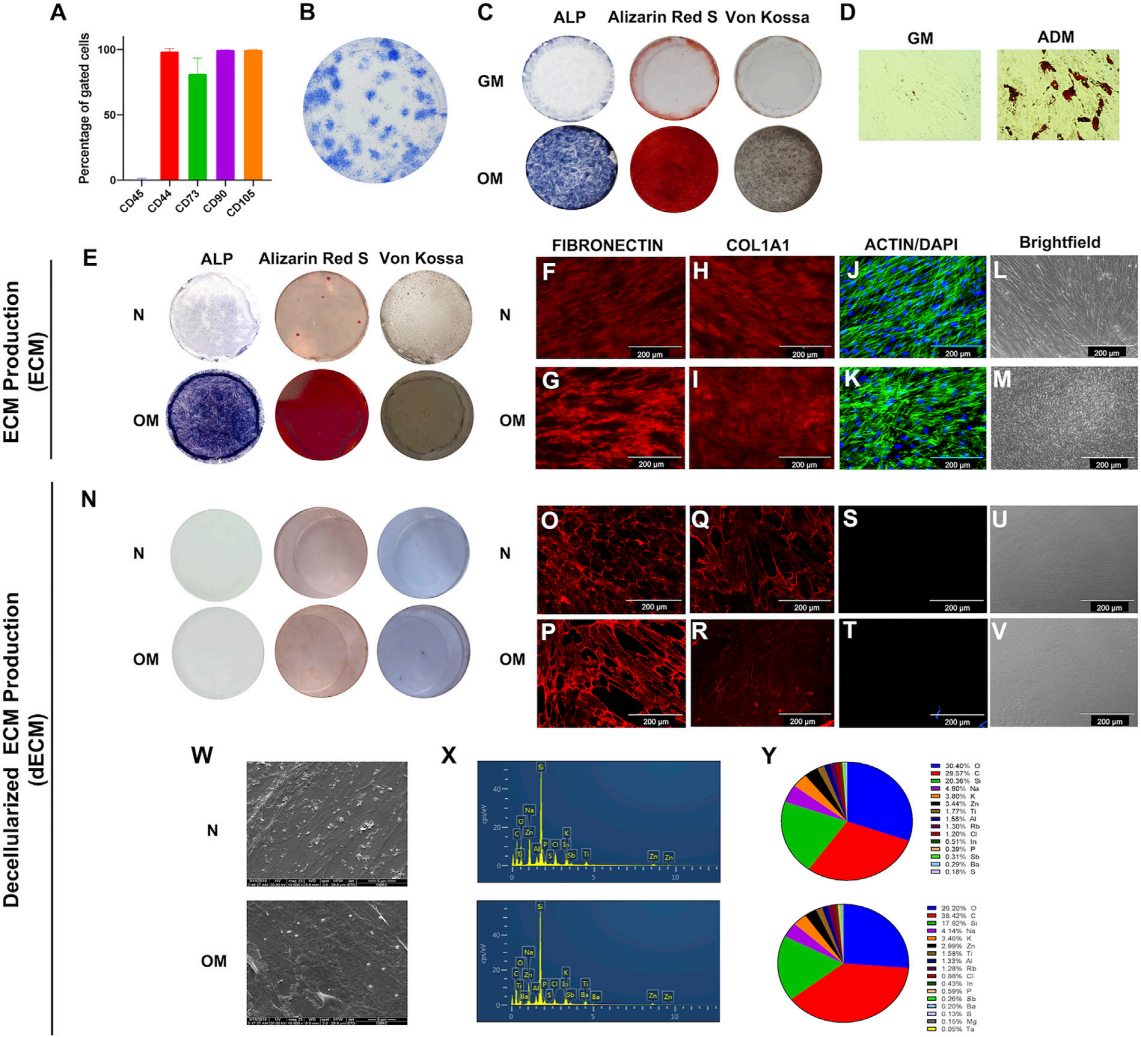
The characterizations of human dental pulp stem cells (DPSCs) and extracellular matrix derived from DPSCs. **(A)** Stem cell surface markers were evaluated by flow cytometry. **(B)** The colony-forming unit ability was examined on day 14 using Coomassie blue staining. Osteogenic and adipogenic differentiation ability were determined at days 14 and 16, respectively. **(C)** ALP activity, calcium and phosphate accumulation at day 14 by staining of BCIP/NBT, Alizarin Red S, and Von Kossa, respectively. **(D)** The intracellular lipid droplets were determined by using oil red O staining. ECM production from DPSCs were investigated. **(E)** ALP activity, calcium nodule formation, and phosphate accumulation were examined using BCIP/NBT, Alizarin Red S, and Von Kossa staining, respectively. **(F**–**I)** Immunofluorescent staining technique used to determine proteins expression of ECM protein including fibronectin and type I collagen. **(J** and **K)** Intracellular cytoskeleton and nucleus was observed using phalloidin and DAPI. **(L** and **M)** Brightfield microscope observations of ECM production. Decellularized ECM (dECM) were characterized. **(N)** ALP and mineral deposition were examined using of BCIP/NBT, Alizarin Red S, and Von Kossa staining, respectively. **(O**–**R)** fibronectin and type I collagen were detected using immunofluorescence staining. **(S** and **T)** Actin filament and DAPI was examined. **(U** and **V)** Brightfield microscope observations of dECM production. **(W)** Cell morphology was observed using scanning electron microscope. **(X** and **Y)** The chemical composition was determined using energy dispersive X-ray analysis, and the percentage of chemical composition was illustrated.

Cells isolated from dental pulp tissue exhibited markers of the mesenchymal stem cell: CD44, CD73, CD90, and CD105, but CD45, a hematopoietic marker, was not detected (Figure 1A). These cells formed colonies when seeded at low density, confirming their self-renewing ability (Figure 1B). The multipotential differentiation ability was determined. Alkaline phosphatase and mineral deposition were increased when cells were cultured in osteogenic induction medium for 14 days (Figure 1C). Quantification of mineralization by alizarin red staining showed that OM-ECM significantly enhanced calcium accumulation compared to N-ECM (Supplementary Figure S2A). Further, the intracellular lipid droplets were noted when cultured in the adipogenic induction medium for 16 days (Figure 1D). In addition, the osteogenic (*ALP*, *OPN*, *OSX*, *RUNX2*, and *OCN*) and adipogenic (*LPL* and *PPARγ*) marker genes expressions were significantly upregulated after osteogenic and adipogenic induction, respectively (data not shown).

To illustrate the characteristic of N-ECM and OM-ECM derived from DPSCs. The accumulation of alkaline phosphatase, calcium, and phosphate was noted in OM-ECM compared to N-ECM (Figure 1E). Moreover, N-ECM and OM-ECM cells produced the ECM proteins: fibronectin (Figures 1F, G) and type I collagen (Figures 1H, I). Actin filaments and nuclei were identified (Figures 1J, K). Cells shapes were observed with a brightfield microscope (Figures 1L, M).

ECM was then decellularized by removing cellular components (dECM). The decellularization process resulted in an ECM without cells and mineral deposits. ALP, Alizarin Red S, and Von Kossa staining were negative in both N-dECM and OM-dECM conditions (Figure 1N). Alizarin Red S quantification confirmed that decellularization process removed mineral deposition (Supplementary Figure S2A). On the other hand, both dECM showed positive ECM protein expression: FN and COL1 (Figures 1O–R). Markedly, no remaining cell was observed as shown by the negative actin and DAPI nuclei staining (Figures 1S, T) as well as by the brightfield microscope observations (Figures 1U, V). These results demonstrated complete removal of cellular component and DNA from ECM samples.

Scanning electron micrographs illustrated ECM fibers after decellularization. Both N-dECM and OM-dECM exhibited similar morphological appearances as well as chemical components as determined by energy dispersive X-ray (EDX) analysis (Figures 1W–Y). Remarkably, OM-dECM did not exhibit the presence of Ca and P components (Figure 1X), confirming the negative Alizarin Red S and Von Kossa staining and the removal of Ca and P after decellularization.

### 3.2 Extracellular Matrix Derived From Dental Pulp Stem Cells Exhibited Matrisome Proteins

**FIGURE 2:**
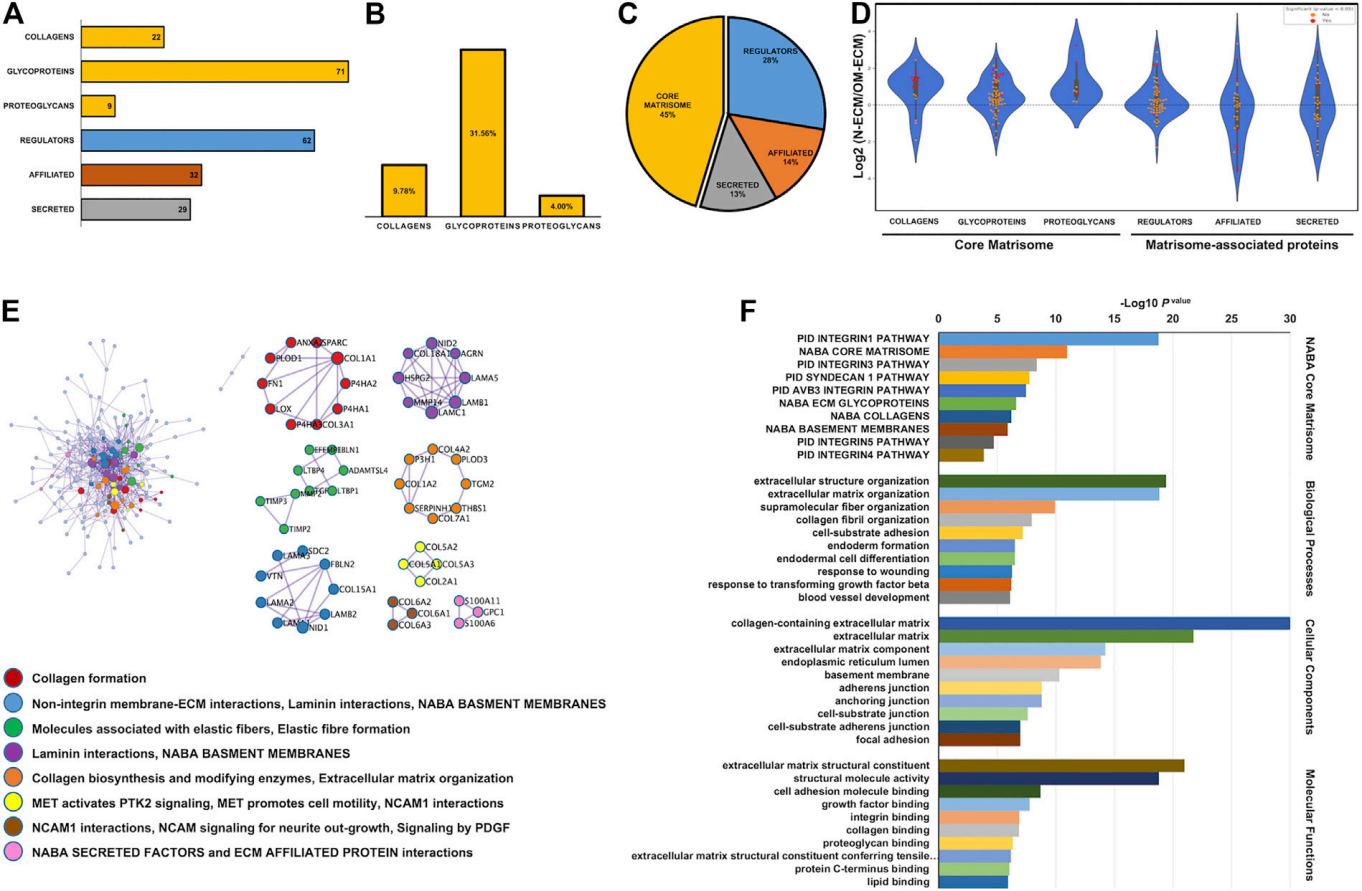
Matrisome analysis of extracellular matrix derived from human dental pulp stem cells (DPSCs). Matrisome categories of the detected total proteins in both normal condition (N-ECM) and osteogenic differentiated condition (OM-ECM) derived from DPSCs by quantitative labelled free proteomics. **(A)** ECM derived from DPSCs in both conditions established the absolute number of different detected matrisome proteins. **(B)** The bar graph showed percentage of core matrisome (collagens, glycoproteins, and proteoglycans) and **(C)** matrisome associate proteins illustrated in pie chart (core matrisome, regulators, affiliated, and secreted). **(D)** Log2 ratio of these proteins obtained from N-ECM and OM-ECM was represented in a violin plot according to the matrisome categories. Red dots represent statistically significant ratios between the two conditions (*p*-value <0.05). Network analysis of protein–protein interaction (PPI) and biological enrichment analysis of ECM derived from DPSCs. **(E)** The diagram showed all high-confidence PPI by metascape analysis and the meanings of network component of PPI using GO enrichment analysis. The nodes color was applied to each protein network. **(F)** Reactome biological pathway and process enrichment analysis. Gene Ontology enrichment analysis of protein involved in NABA matrisome pathway, cell component, molecular function, and biological process. The bar plot showed the enrichment scores (−log10 [*p*-value]) of the significant difference.

N-ECM and OM-ECM proteins from cellular compartment isolation were analyzed. Matrisome proteins were composed of 225 individual different proteins according to Human Matrisome Database (http://matrisomeproject.mit.edu/other-resources/human-matrisome/) (Figure 2A and Supplementary Table S2). Detected proteins were classified according to different categories, the three core matrisome categories—9.78% of collagens, 31.56% of glycoproteins, and 4.00% of proteoglycans—and the matrisome-associated protein categories—28% of regulators, 14% of affiliated, and 13% of secreted proteins (Figures 2B, C). The proteins ratios were detected by quantitative label-free proteomics analysis in the N-ECM and OM-ECM. The significant differences between two conditions are represented through red dots on violin plots in the log2 ratio of N-ECM compared to OM-ECM (Figure 2D). Core matrisome proteins are mostly upregulated in N-ECM, while matrisome-affiliated proteins are in OM-ECM (Figure 2D).

The protein–protein interactions (PPI) of ECM derived from DPSCs contained 54 protein complex (clusters) from 188 nodes and 592 edges (Figure 2E). Gene Ontology (GO) enrichment analysis was applied to each network component. Results showed the interaction network of collagen formation, integrin membrane interaction, elastic fiber formation and laminin interaction, the activation of focal adhesion MET receptor, and the interaction of neural cell adhesion molecule (NCAM). In addition, GO functional enrichment analysis was performed; ECM derived from DPSCs were categorized in NABA core matrisome, cellular component, biological process, and molecular function, respectively (Figure 2F). The most significant were found in the collagen-containing ECM, ECM structural constituent, extracellular structure organization, and integrin 1 pathway. These enriched pathways confirm the mesenchymal origin of the dental pulp cells and the participation of DPSC to pulp ECM formation.

### 3.3 Extracellular Matrix Derived From Dental Pulp Stem Cells Exhibited Specific Matrisome Protein of Elastic Fiber in L-Ascorbic Acid and Affiliated Proteins in Osteogenic Induction Medium

**FIGURE 3:**
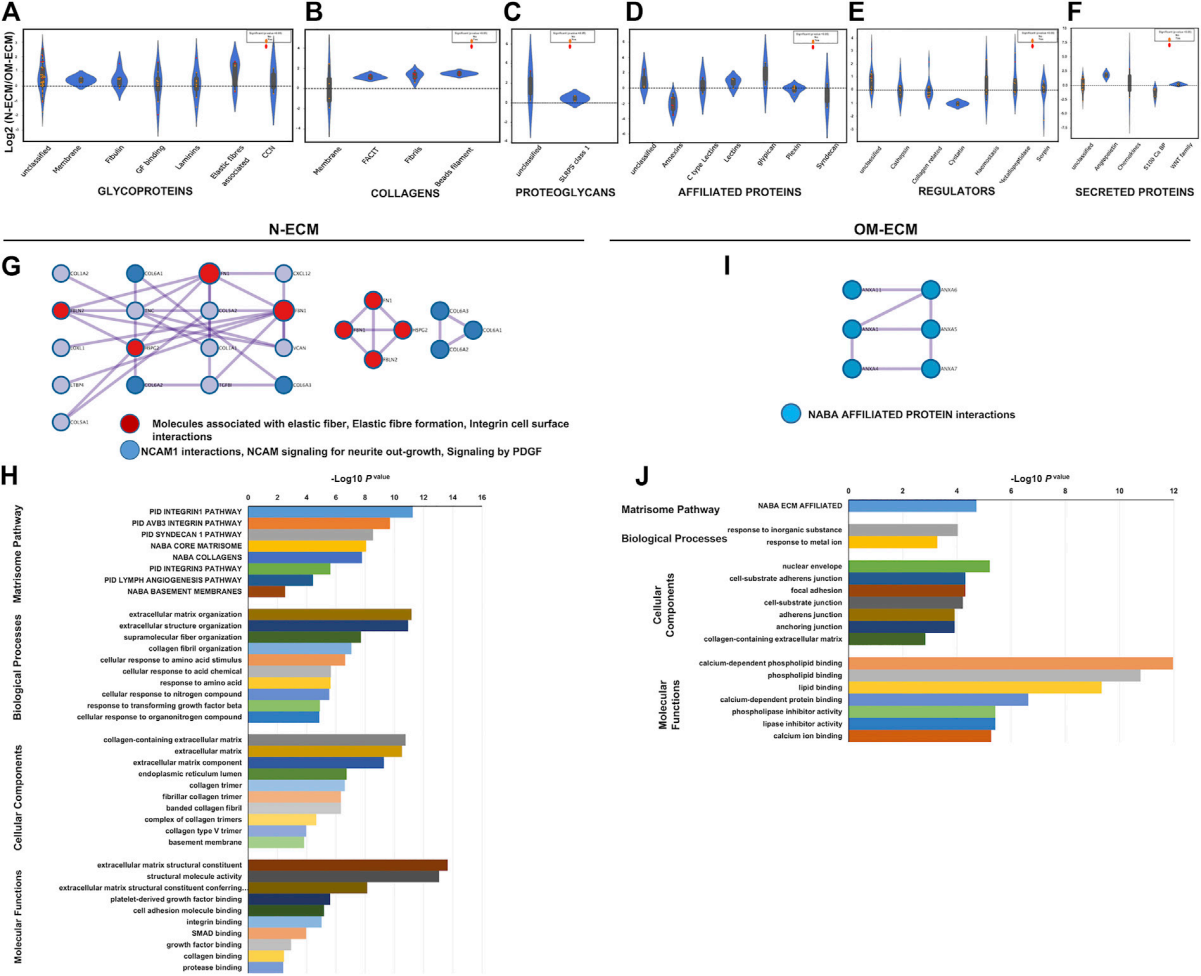
Matrisome analysis of extracellular matrix derived from human dental pulp stem cells (DPSCs) revealed differences between N-ECM and OM-ECM. Log2 ratio of the matrisome proteins obtained from N-ECM and OM-ECM were represented in a violin plot including core matrisome; **(A)** glycoproteins, **(B)** collagens, and **(C)** proteoglycans. Matrisome-associated proteins; **(D)** affiliated proteins, **(E)** regulator, and **(F)** secreted proteins. Red dots represented statistically significant ratios between the two conditions (*p*-value <0.05). Network analysis of protein–protein interaction (PPI) and biological enrichment analysis of N-ECM **(G** and **H)** and OM-ECM **(I** and **J)** was demonstrated. The diagram showed all high-confidence PPI by metascape analysis and the meanings of network component of PPI using GO enrichment analysis. The nodes color was applied to each protein network, reactome biological pathway and process enrichment analysis, Gene Ontology enrichment analysis of protein involved in NABA matrisome pathway, cell component, molecular function, and biological process. The bar plot showed the enrichment scores (−log10 [*p*-value]) of the significant difference.

N-ECM significantly increased some core matrisome proteins; meanwhile, OM-ECM produced overdetected associated matrisome proteins significantly (Figures 3A–F). Differentially expressed proteins are listed in Supplementary Table S2. In addition, the significant ratios upregulated in N-ECM represented collagen proteins including fibrillar (COL1A1, COL1A2, COL2A1, COL3A1, COL5A1, COL5A2, and COL5A3), fibril-associated collagen with interrupted triple helixes (FACIT) (COL12A1, COL14A1, COL16A1, and COL22A1), and beads filament (COL6A1, COL6A2, and COL6A3). Moreover, glycoproteins including elastic fiber-associated (EMILN1 and FBN1), fibulin (FBLN2), growth factor binding (LTBP4), laminin (LAMA3), major glycoprotein (FN1, MXRA5, and TNC), unclassified protein (POSTN, TGFBI, and THSD4), and proteoglycans (VCAN, HSPG2) were significantly identified. In OM-ECM, the significant overdetected matrisome proteins were affiliated proteins including annexins (ANXA1, ANXA4, ANXA5, ANXA6, ANXA7, and ANXA11). Further, glycoprotein (elastic fibers; FBN2, growth factor binding; IGFBP4, and unclassified protein; CRELD2) and matrisome-associated regulator protein (collagen related; PLOD3) were significantly indicated.

Accordingly, PPI of N-ECM matrisome proteins were composed of 7 clusters from 17 nodes and 23 edges and showed protein complexes of elastic fiber and NCAM interaction (Figure 3G). GO enrichment identified abundances of integrin1 pathway, ECM organization, collagen-containing ECM, and ECM constituent (Figure 3H). Interestingly, PPI of OM-ECM matrisome proteins contained 6 nodes and 6 edges (Figure 3I). The network detected NABA-affiliated protein interactions; GO enrichment analysis showed enrichment of calcium-dependent phospholipid binding in molecular function (Figure 3J).

### 3.4 Decellularized Extracellular Matrix From Dental Pulp Stem Cells Enhanced Osteogenic Differentiation and Mineralization Potency of Gingival Fibroblasts

**FIGURE 4:**
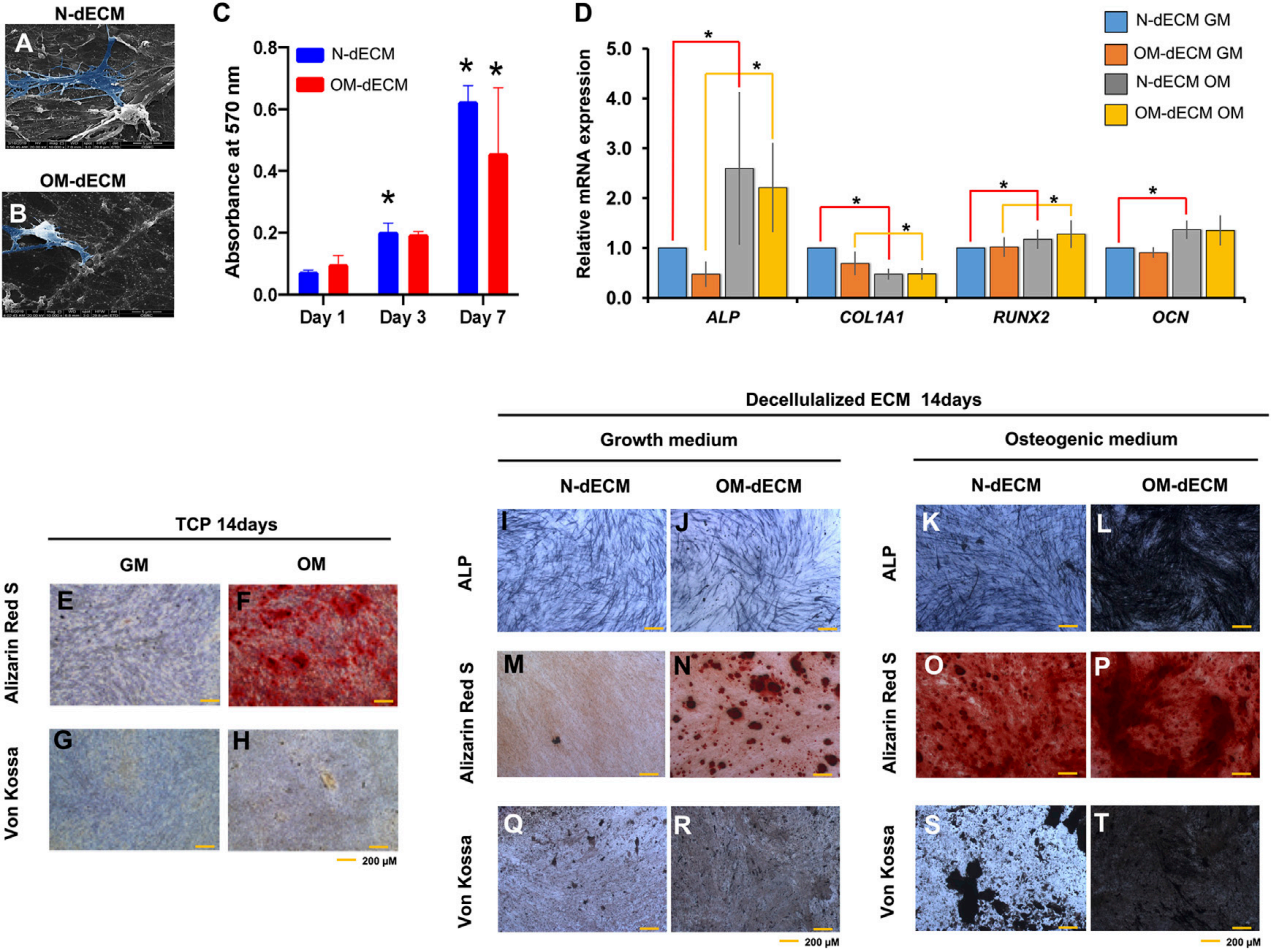
Decellularized extracellular matrix (dECM) from human dental pulp stem cells (DPSCs) enhanced osteogenic differentiation potency of gingival fibroblasts (GF). GF were reseeded on N-dECM and OM-dECM cultured with growth medium or osteogenic differentiation medium. **(A** and **B)** Cell attachment was examined at 24 h using a scanning electron microscope analysis. **(C)** Cell metabolic activity was examined using MTT assay on days 1, 3, and 7. **(D)** The mRNA expression of osteogenic marker gene was evaluated using real-time quantitative PCR. GF were seeded on TCP; after osteogenic differentiation for 14 days, ALP staining and mineral accumulation were determined using BCIP/NBT and Alizarin Red S and Von Kossa staining, respectively **(E–H)**. GF were reseeded on N-dECM or OM-dECM and subsequently cultured in growth medium **(I–R)** or osteogenic induction medium **(K–T)**. Cell seeded on TCP were used as control. Asterisks indicate a statistically significant difference compared with the control (*p*-value <0.05).

To examine the osteogenic differentiation ability of GF on decellularized ECM derived from DPSCs, GF were seeded on N-dECM and OM-dECM. After 24 h, GF were able to attach and spread on both N-dECM and OM-dECM (Figures 4A, B). GF survived on N-dECM and OM-dECM maintained under growth medium condition. Moreover, an increase in GF metabolic activity was observed from days 1–7 (Figure 4C). GF cultured for 1, 3, or 7 days showed no differences in proliferation when cultured either on TCP or N-dECM or OM-dECM (Supplementary Figure S1).

For osteogenic differentiation potential of GF on dECM, GF were seeded on N-dECM and OM-dECM and subsequently maintained in growth medium or osteogenic induction medium for 14 days. GF reseeded on N-dECM (red line) and cultured in osteogenic induction condition showed the significant upregulated *ALP*, *RUNX2*, and *OCN* and downregulated *COL1A1* mRNA expression (Figure 4D). GF reseeded on OM-dECM (yellow line) upregulated *ALP and RUNX2* and downregulated *COL1A1* mRNA expression. There was no statistically significant difference of osteogenic marker gene expression between GF reseeded on N-dECM and OM-dECM under growth medium.

The mineral deposition of GF was highly enhanced on dECM compared to TCP at day 14 (Figures 4E–H). ALP expression was markedly increased in GF reseeded on OM-dECM maintained in osteogenic induction medium compared to other conditions (Figures 4I–L). Similarly, calcium and phosphate accumulation were markedly increased in OM-dECM condition (Figures 4M–T). Interestingly, GF on OM-dECM significantly enhanced calcium and phosphate deposition even in growth medium (Figures 4N, R). Quantification of mineralization by alizarin red staining was performed; the absorbance of the solubilized Alizarin Red S dye was measured at 570 nm (Supplementary Figures S2B, C). This confirmed the increase of mineral deposition in OM-dECM of GF cultured in normal medium compared to N-dECM.

## 4 Discussion

The present study identifies and characterizes the composition and functional differences of ECM derived from DPSCs. In this study, we showed ECM derived from DPSCs exhibited classical ECM protein expression (type I collagen and fibronectin) whatever the culture media. Moreover, DPSC produced a diverse ECM where some matrisome proteins differ according to culture medium albeit the DPSC matrisome composition remains almost the same. Calcium and phosphate deposition within ECM confirmed the osteogenic differentiation and was associated to osteo-inductive conditions.

The aim of decellularization process was to remove cellular components from cell-derived ECM. After decellularization, we detected neither filamentous actin nor nuclei. DNA within dECM significantly lowers after decellularization (Guneta et al., 2017; Jeon et al., 2018). This confirms our results; the decellularizing processes effectively removed both cell components and mineral deposition in ECM as evidenced by negative staining with DAPI, Alizarin Red S, and Von Kossa in dECM. Moreover, the Ca and P components in EDX analysis were not prominent in both N-dECM and OM-dECM, confirming the demineralizing effect.

According to ECM classified by matrisome analysis (Naba et al., 2016), the present study identifies and quantifies the ECM proteins derived from DPSCs. We found 225 matrisome proteins showed highest abundance from glycoproteins, then regulator proteins, affiliated proteins, secreted proteins, collagens, and proteoglycan, respectively. On the other hand, 146 proteins were found in matrisome proteins of ECM derived from dentin and detected collagens, glycoprotein, affiliated proteins, regulator proteins, secreted protein, and proteoglycans, respectively (Reis et al., 2020). ECM of periodontal ligament (another oral tissue) is composed of 105 matrisome proteins, and highest detection was of regulator proteins, then glycoproteins, collagens, affiliated proteins, proteoglycans, and secreted proteins, respectively (Denes et al., 2020). Therefore, differences among ECM proteins in each matrisome component might be specific of a type of tissue and might lead to specific regulation and function from ECM proteins.

Biological network analysis is a powerful approach to achieve insights into biological systems. The interactions of different proteins regulate the mechanisms associated with physiology and pathology (Gonzalez and Kann 2012). The most significant functional enrichment in DPSCs matrisome is cellular component of collagen-containing ECM, correspondingly with PPI to collagen formation. Dental pulp is a connective tissue, which is composed of collagen and elastic fibers and contains glycoproteins such as fibronectin and laminins (Yildirim 2013). We confirmed that DPSCs produce these ECM proteins and thus behave like mesenchymal cells. Moreover, the PPI of matrisome pathways showed integrin *β*_1_ surface and laminin interactions. Correspondingly, these biological functions showed regulated DPSCs attachment to ECM (Zhu et al., 1998). For this reason, integrin–laminin interaction on ECM might be a major biology function of DPSCs attachment. About cell adhesion, our data confirmed the detection of fibronectin (together with integrin *β*_1_) which is necessary for odontoblasts differentiation (Lesot et al., 1981; Lesot et al., 1990; Saito et al., 2015). NCAM signaling for neurite out-growth pathway is important for the formation and maintenance of the nervous system of cellular processes (Panicker et al., 2003), which is found in DPSCs matrisome, especially in normal matrisome (N-ECM). It is possible that DPSCs, which originated from a cranial neural crest lineage, retain a remarkable potential for neuronal differentiation or induction and additionally express multiple factors that are suitable for neural or axonal regeneration (Luo et al., 2018).

Osteogenic differentiation/mineralization may be driven by secreted factors such as insulin growth factor (IGF) and IGF binding proteins, which were measured in the DPSCs ECM (IGFBP2, IGFBP4, IGFBP5, IGFBP5, and IGFBP7). Moreover, transforming growth factor (TGF) beta pathway appears to be regulated by some of the detected ECM proteins (Supplementary Figure S3). Last, we know that cells binding to substrate also regulate cells fate, so integrin binding and focal adhesion may also impact these cells' behavior. Otherwise, in OM-dECM, annexins may play a role in the increased ability to induce mineralization as they appeared to be enriched and are known mineralization factors (Cmoch et al., 2011; Genetos et al., 2014; Pan et al., 2015).

The present study showed that annexins are enriched in OM-ECM vs. N-ECM. We hypothesize that annexins could participate in the osteogenic inductive properties of OM-dECM derived from DPSCs. A positive role of annexins on osteogenic differentiation and ossification has been reported (Grewal et al., 2021). ANXA1, ANXA4, ANXA5, ANXA6, ANXA7, and ANXA11 were enriched in OM-ECM compared to N-ECM in the present study. Rat bone marrow MSC transfected with shRNA against annexin1 exhibited a reduction of mineralization *in vitro* and osteogenic marker gene expression (Pan et al., 2015). Annexin A2 or A5 knockdown in murine pre-osteoblast cell line resulted in the dramatic decrease of alkaline phosphatase enzymatic activity, while mineral deposition and osteogenic marker gene expression were not robust (Genetos et al., 2014). Annexins A1, A6, A7, and A11 were associated with S100 proteins, especially S100-A6 and S100-A10, in mineralizing matrix vesicles from osteoblasts (Cmoch et al., 2011). Consistent with the present study, annexin and S100 proteins were detected in DPSCs-derived OM-ECM; however, the S100 proteins ratio are not considered significantly different from N-ECM. These data support our hypothesis regarding the role of increased annexins in OM-ECM on its mineralization inductive properties. Therefore, the influence of annexins on ECM derived from DPSCs in mineralization requires further investigations.

On the other hand, the collagens and glycoproteins were enriched in N-ECM compared to OM-ECM. These proteins are classically associated with connective soft tissue matrix, such as elastic fibers or non-fibrillary collagens (COL6). Correspondingly, biological functions of OM-ECM showed an enrichment of inorganic substances and NABA-affiliated protein interactions, whereas N-ECM was enriched with elastic fibers and integrin interactions. We also demonstrated an over-representation of classical fiber proteins in N-ECM: fibril forming, FACIT, and beaded collagens together with some major ECM glycoproteins such as fibronectin. This confirmed the ECM proteins differences N-ECM and OM-ECM corresponded to the usual soft tissue and mineralization matrix. It highlights the ability of DPSCs to produce different ECM and the similarities between *in vivo* conditions and our experiments. However, it is also noteworthy that most of the ECM proteins are not differentially detected; the biological properties can be modified through few ECM changes, meaning the great impact of dental pulp biological functions.

CXCL12, also known as stromal cell-derived factor 1 (SDF1), is a chemokine protein that functions as a chemotactic factor for mesenchymal stem cells and is expressed in areas of inflammatory bone destruction, where it mediates their suppressive effect on osteoclastogenesis (Takano et al., 2014) as well as an important role in angiogenesis (Zhang and He, 2019). On the other hand, Soluble frizzled-related proteins (sFRPS) modulate Wnt signaling by direct interaction with Wnts and secreted frizzled-related protein 1 (SFRP1). They play a part in controlling cell development and differentiation in certain cell types. Wnt signaling components have positive impacts on pulp healing, dentin repair, and stemness maintenance epigenetic control (Yaemkleebbua et al., 2019; Kornsuthisopon, 2022). SFRP1 is a Wnt antagonist that acts as an ECM inhibitor of the Wnt signaling pathway, preventing osteoblast-induced osteoclastogenesis and maintaining the nonmineralized state of PDL progenitors (Hausler et al., 2004; Gopinathan et al., 2019). Therefore, the overexpression of CXCL12 and SPFR1 on N-ECM could be attributable to the regulation of DPSC homeostasis in the nonmineralized condition. Indeed, the signaling pathway regulates dental pulp homeostasis, and dentin regeneration required further investigation.

An ECM derived from cell culture is a natural biomaterial. This potential scaffold is free of cellular components, while retaining the complex network of both structure and functional protein assembled in their environment. Under chemically controlled settings, cell-derived extracellular matrices have the potential to be utilized as tissue replacements in a relatively short period of time (Ahlfors and Billiar 2007). Decellularization is completed by eliminating genetic material with DNase to reduce host immunological reactivity, as seen in tissue-derived ECMs (Crapo et al., 2011). Thus, in the future, cell culture-based ECMs could be considered as an innovative technique for direct differentiation of even somatic cells into other cell types, according to the findings of this study. Correspondingly, decellularization of natural ECM derived from cell culture showed potential in cellular biological properties such as cell proliferation and gene expression as well as cell remodeling and differentiation (Parmaksiz et al., 2020). However, dECM derived from cells as a two-dimensional (2D) structure lacks mechanical strength and stability. Hybridized decellularization with crosslinking agents, synthetic polymers, or hydrogels might be necessary to improve dECM scaffolds towards increased efficiency and higher innovation of natural biomaterial to be used in regenerative medicine (Nouri Barkestani et al., 2021).

dECM derived from tissues has shown the potential to be used as regenerative materials, for example, in pulp therapy (Bakhtiar et al., 2020). Furthermore, dentin ECM components promoted DPSC mineralization (Petridis et al., 2019). Thus, decellularized ECM derived from tissue or cells requires further investigation.

GF were selected as a model for reseeding on dECM in order to determine the regulation of DPSC-derived ECM on osteoblastic differentiation and mineralization induction. GF are the most abundant oral cell types that could be isolated from patients. The tissue availability and non-invasive harvesting procedures support the use of GF in regenerative dentistry (Fournier et al., 2013). They present less osteogenic differentiation potency compared with other dental mesenchymal stem cells (Dan et al., 2014). Therefore, GF were suitable to observe the putative dECM inductions on osteogenic differentiation and mineralization. Reseeded cells on dECM derived from DPSCs in different culture media did not affect cell viability responses. Both N-dECM and OM-dECM did not affect GF cell viability. This result is similar to the study in hADSC showing that there was no significant alteration of cell proliferation on dECM (Guneta et al., 2017). On the contrary, dECM derived from bone marrow increased periodontal ligament stem cells (PDLSCs) number compared to dECM derived from PDLSCs (Wen et al., 2019). These results imply that DPSCs ECM derived from different induction conditions do not markedly influence the cell proliferative function.

OM-dECM derived from DPSCs dramatically promoted osteogenic differentiation of GF compared to N-dECM in the presence or absence of osteogenic inductive factors. *COL1A1* mRNA expression was downregulated in both N-dECM and OM-dECM at mineralization state. Col1a1 messenger RNA (mRNA) is an early marker during osteogenic differentiation appearing after mineralization process (Dacic et al., 2001). Likely, the osteogenic marker genes expression of GF in N-dECM showed upregulated *ALP*, *RUNX2*, and *OCN.* The upregulation of *ALP* and *RUNX2* were expressed in GF on OM-dECM but not significantly different when cultured under different environments. Correspondingly, dECM derived from differentiation osteoblast showed more effective osteogenic differentiation than growth medium-derived dECM (Jeon et al., 2018).

The Alizarin Red S and Von Kossa staining on N-dECM and OM-dECM confirmed that OM-dECM was sufficient to induce mineralization. Interestingly, reseeded GF on dECM enhanced mineralization faster than that cultured on TCP at day 14. “Besides, mineralization, at 14 days under osteogenic induction condition, was significant in both N-dECM and OM-dECM. No differences were observed at this timepoint between conditions. However, observations along time might reveal mineralization differences between those groups” (Misof et al., 2020).

Taking all data together, the results implicate that the mineralization of GF can be induced by OM-dECM, which may be linked to specific proteins from OM-ECM. Thus, OM-dECM is mineralization-inductive.

## 5 Conclusion

**FIGURE 5:**
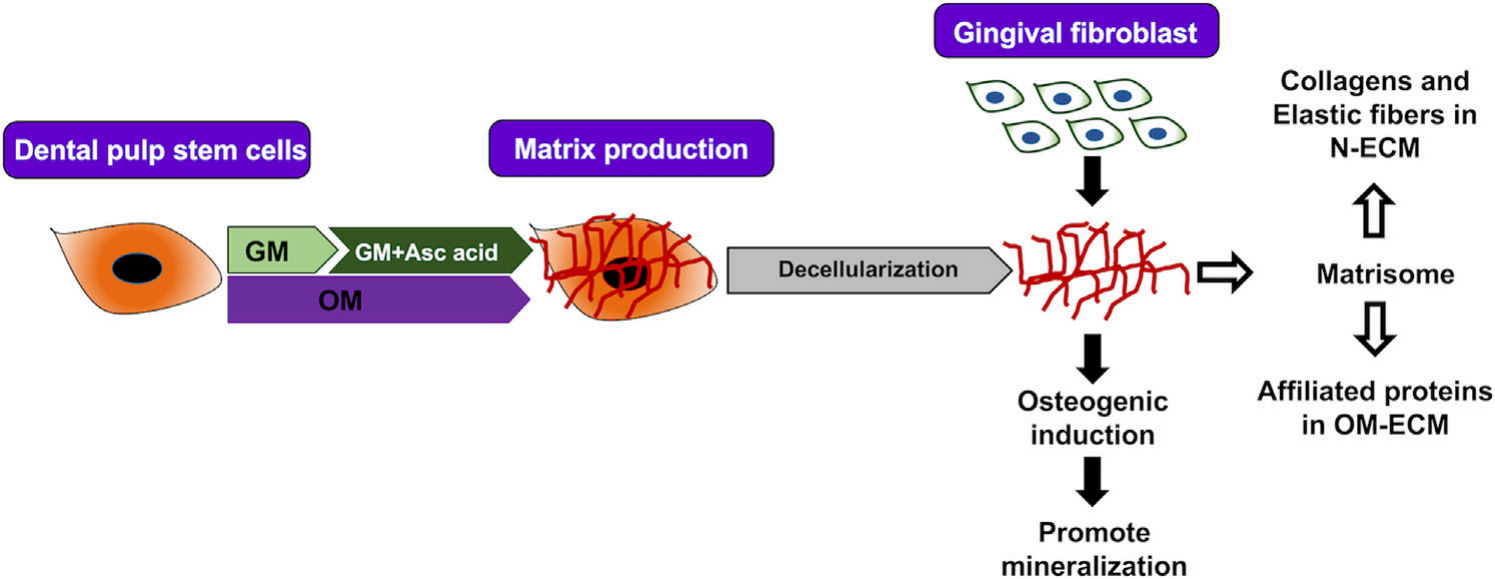
Summary. Extracellular matrix (ECM) from human dental pulp stem cells was produced by two conditions (N-ECM—normal medium supplement with ascorbic acid, and OM-ECM—osteogenic induction medium) for 21 days. ECM in normal and mineralized conditions enhance mineralization of gingival fibroblasts after reseeding on ECM. Proteomic analysis of ECM was defined by matrisome database. Normal conditions ECM (N-ECM) shows enrichment of collagens and elastic fibers formation. On the other hand, mineralized conditions ECM (OM-ECM) shows enrichment of matrisome-affiliated proteins.

The present study shows that matrisome proteins of DPSCs-derived ECM differ according to culture conditions, with increased core matrisome proteins (collagens, elastic fibers associated) in N-ECM, while matrisome-associated proteins (annexins) were enriched in OM-ECM. We demonstrated that ECM impacts cell behavior and differentiation. DPSCs ECM proved to be mineralization inductive (Figure 5). Our identified ECM proteins can be used as a marker for osteogenic differentiation. Further study will investigate the patterns of those proteins during osteogenic differentiation. This *in vitro* ECM or some of its proteins may be helpful for tissue engineering and could be used for mineralized tissue therapy or to decorate biomaterials.

### 6 Limitations

Quantities of peptides before mass spectrometry might be lost during sample processing. Some of the significant differences should be interpreted with caution. Furthermore, the sensitivity of the mass spectrometer substantially limited the detection threshold for protein identification in this study. (*q*-value <0.01). Potentially, interesting proteins with lower expression levels may have been underestimated. Therefore, proteins that are identified as unique in N-ECM may also be expressed in very low quantities in OM-ECM or vice versa.

## Data Availability

The datasets presented in this study can be found in online repositories. The names of the repository/repositories and accession number(s) can be found below: PRIDE database, accession no: PXD018951.
