# Supplementary figures and images for: Extracellular Matrix Derived From Dental Pulp Stem Cells Promotes Mineralization
Source: Front Bioeng Biotechnol. 2022 Jan 27;9:740712. doi: 10.3389/fbioe.2021.740712 (PMC8829122; doi:10.3389/fbioe.2021.740712)

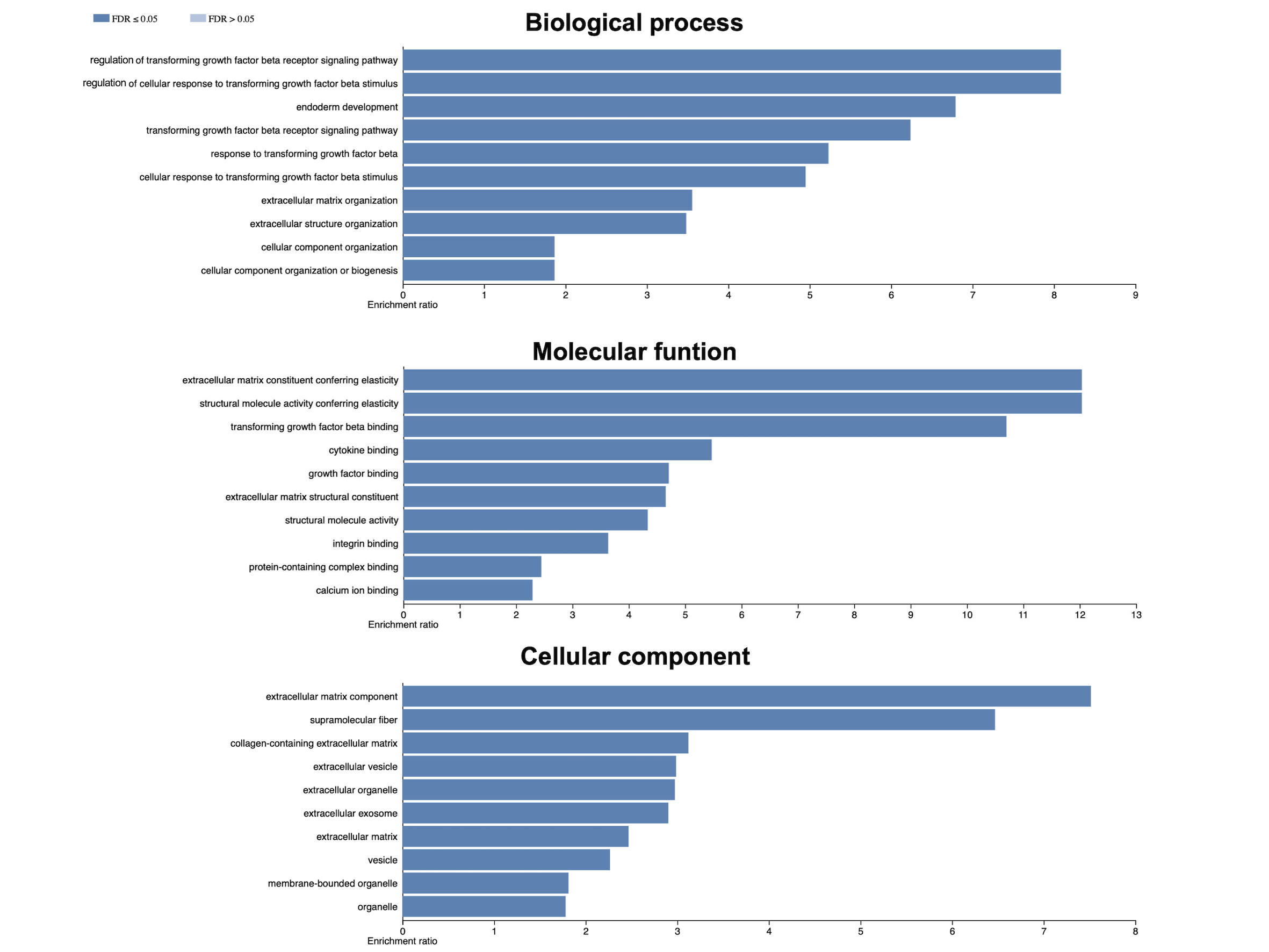

Supplement: Supplementary file 1 [file Image3.TIFF]

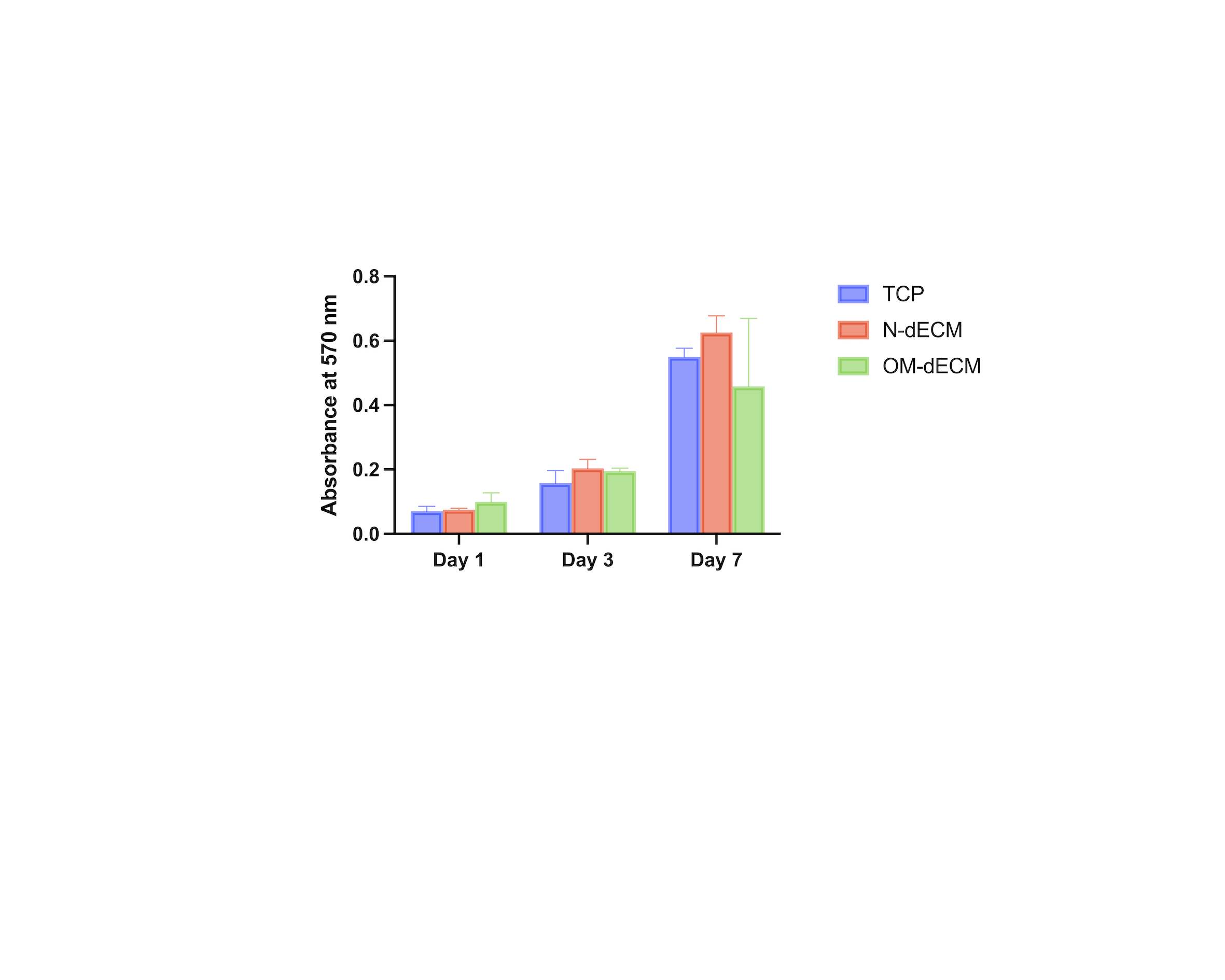

Supplement: Supplementary file 3 [file Image1.TIFF]

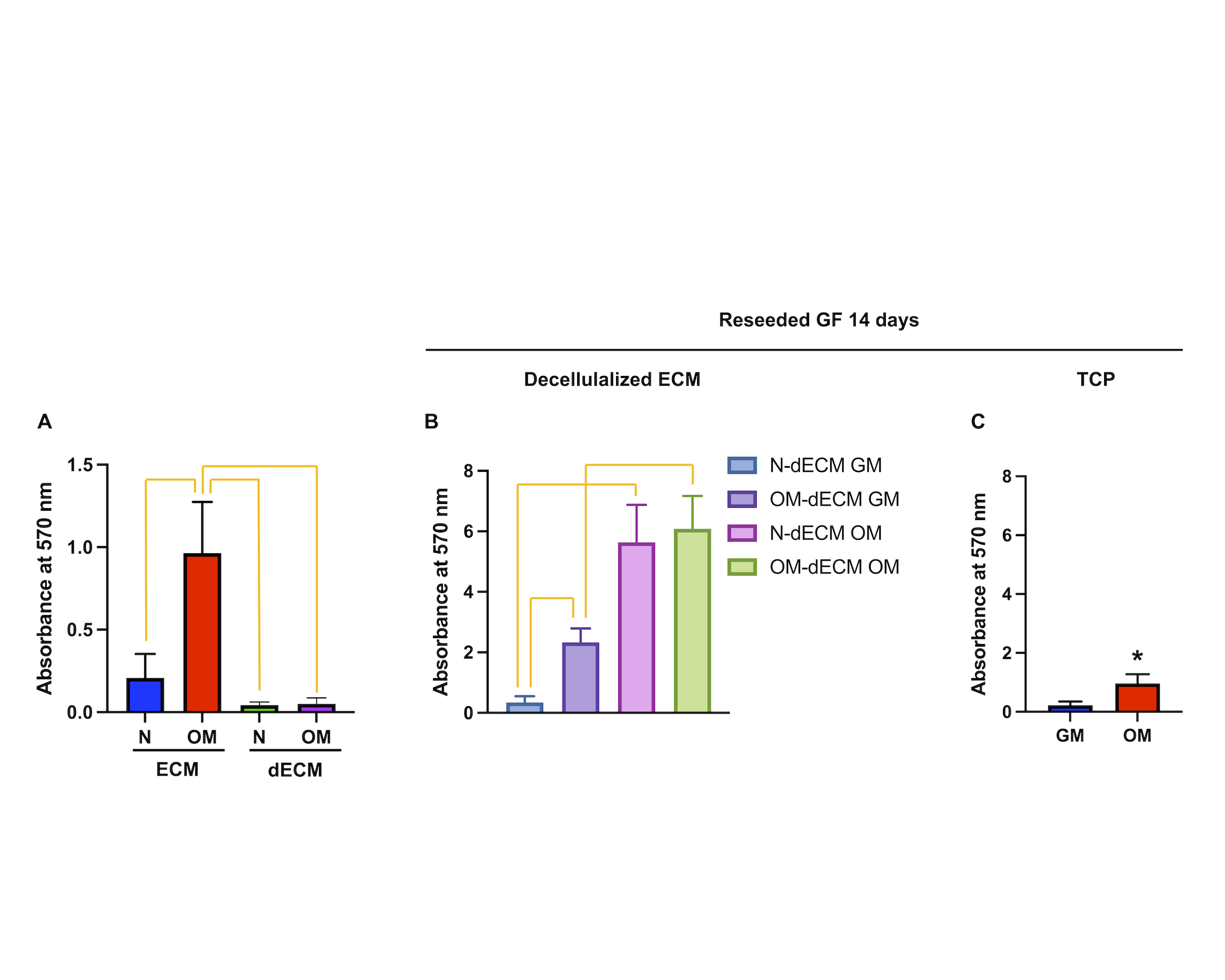

Supplement: Supplementary file 5 [file Image2.TIFF]
