# Supplementary material for: Extracellular Matrix Derived From Dental Pulp Stem Cells Promotes Mineralization
Source: Front Bioeng Biotechnol. 2022 Jan 27;9:740712. doi: 10.3389/fbioe.2021.740712 (PMC8829122; doi:10.3389/fbioe.2021.740712)
Supplement: Supplementary file 4 [file Table1.DOCX]

# Supplementary Tables

## Supplementary Table 1. Primer sequences

| **Gene** | **Forward** | **Reverse** | **Accession number** |
| --- | --- | --- | --- |
| *SDHA* | 5’-GGCGTCCCCCAACTTCTTA-3’ | 5’- TAATCGTACTCATCAATCCG-3’ | NM_004168.4 |
| *TBP* | 5’-CACGAACCACGGCACTGATT-3’ | 5’-TTTTCTTGCTGCCAGTCTGGAC-3’ | NM_003194.5 |
| *ALP* | 5’-CGAGATACAAGCACTCCCACTTC-3’ | 5’-CTGTTCAGCTCGTACTGCATGTC-3’ | NM_000478.3 |
| *COL1A1* | 5’-GTGCTAAAGGTGCCAATGGT-3’ | 5’-ACCAGGTTCACCGCTGTTAC-3’ | NM_000088.3 |
| *RUNX2* | 5’-ATGATGACACTGCCACCTCTG 3’ | 5’-GGCTGGATAGTGCATTCGTG 3’ | NM001024630.3 |
| *OCN* | 5’-CTTTGTGTCCAAGCAGGAGG-3’ | 5’-CTGAAAGCCGATGTGGTCAG-3’ | NM_199173.4 |
