# Supplementary material for: Extracellular Matrix Derived From Dental Pulp Stem Cells Promotes Mineralization
Source: Front Bioeng Biotechnol. 2022 Jan 27;9:740712. doi: 10.3389/fbioe.2021.740712 (PMC8829122; doi:10.3389/fbioe.2021.740712)
Supplement: Supplementary file 6 [file DataSheet1.docx]

Supplementary Material

# Supplementary Methods

## Isolation and expansion dental pulp cell and gingival cell

Teeth or gingival tissue were obtained from surgical treatment from healthy adult patients according to their treatment plan at Department of Oral and Maxillofacial Surgery, Faculty of Dentistry, Chulalongkorn University. The protocol was approved by Human Research Ethic Committee (approval number 079/2018 from Faculty of Dentistry Chulalongkorn University). Cell isolation protocol was performed as previously described (Fournier et al. 2016; Osathanon et al. 2011). Briefly, pulp tissue and gingival tissue were chopped into small pieces and put in 35 mm culture dishes. cells were maintained in growth medium (Dulbecco’s modified eagle medium; DMEM) supplemented with 10% of fetal bovine serum, 100 U/ml of penicillin, 2 mM of L-glutamine, 5 ug/ml of amphotericin B and 100ug/ml of streptomycin in 100% humidity, 37^o^C and 5% carbon dioxide and growth medium were changed every 48 h. after 100% confluence, cells were sub-cultured at 1:3 ratio and cells at passage 4-7 were used in experiments.

Osteogenic and adipogenic differentiation were performed according to previous reports (Fournier et al. 2016; Osathanon et al. 2011). Cells were seeded in 24-wells-plate at density of 50,000 cells per well. At 24 h after seeding, culture medium was changed into differentiation medium. Osteogenic induction medium consisted of growth medium supplemented with 50 µg/mL ascorbic acid, 10 nM dexamethasone and 5mM β‐glyceraphosphate. Adipogenic medium was growth medium using DMEM high glucose supplemented with 10 μg/ml insulin, 1 μM dexamethasone, 500 μM IBMX, 50 μM indomethacin and 50 µg/mL ascorbic acid. Cells cultured in growth medium were used as the negative control.

## Flow cytometry

Cells were trypsinized with 0.25% trypsin-EDTA and washed with PBS containing 1% FBS. Cells were then incubated for 15 mins at 4^o^C in PBS contain 1% FBS with primary antibodies were FITC-conjugated anti-human CD44 mAb (Immunotools, Germany) and anti-human CD73 mAb (Immunotools, Germany), APC‐ conjugated anti‐CD90 antibody (Immunotools, Germany), PE-conjugated anti-human CD105 mAb (Immunotools, Germany), PerCP‐conjugated anti‐CD45 antibody (Immunotools, Germany). Cell analysis was performed on a FACS-Calibur using the CellQuest software (BD Bioscience, USA).

## Colony forming unit assay

Five hundred cells were seeded in 60 mm tissue culture dishes and maintained in growth medium. Culture medium was changed every 48 hrs. At 14 days, cells were fixed with 4% paraformaldehyde (PFA) and stained with coomassie blue. Colony formation was observed under light microscope.

## Alkaline phosphatase staining

Specimens were fixed with 4% PFA for 10 mins at room temperature and then incubated with BCIP/NBT tablets (Roche, USA) for 30 mins at room temperature under dark condition. Samples were then washed 3 times with deionized water. The colored under light microscope.

## Mineralization assay

For Alizarin Red S staining, cells were fixed in 4% PFA for 10 mins. After washed with deionized water, the cells were stained with 1% Alizarin red S (Sigma, USA) solution at room temperature. Each of the stained specimens was washed by deionized water and then air-dried. For Von Kossa staining, cells were incubated with 1% silver nitrate (Sigma, USA) solution for 20 mins after fixed with cold methanol. The incubation was performed under UV light. After rinsing with deionized water, samples were then incubated with 5% sodium thiosulfate for 5 mins and subsequently washed with deionized water and air-dried. The colored was observed under light microscope. The quantification was determined by eluting staining with 10% w/v cetylpyridinium chloride solution in 10 mM sodium phosphate and the absorbance was measured at 570 nm.

## Intracellular lipid staining

Specimens were fixed in 4% PFA for 10 mins. After washed with PBS, the specimens were then incubated with 60% isopropanol for 5 mins and subsequently stained with Oil Red O (Sigma, USA) solution for 5 mins at room temperature, then rinsed with 60% isopropanol.

## Immunofluorescence staining

Specimens were fixed in 4% PFA and permeabilized with 0.1% Triton-X100. Unspecific binding proteins was blocked with 0.1% BSA in PBS for 30 mins. The primary antibodies (mouse monoclonal IgG anti Collagen 1, Abcam, USA, mouse monoclonal IgG anti fibronectin, DSHB, USA) were prepared in 0.1% BSA in PBS. Specimens were incubated with primary antibodies for 2 hrs at room temperature. The secondary antibody labeled with AlexaFluor 488, AlexaFluor 594 was added at 1:500 dilution for 45 mins. DAPI counterstained the nuclei. Actin filaments were observed using AlexaFluor 488 Phalloidin. The fluorescence was evaluated by fluorescence microscope (Apotome).

## Extracellular matrix preparation

Tissue culture plate (TCP) surface was coated with 0.2% gelatin for 2 hrs at 37^o^C. Coated plate washed 2 times with sterile PBS and incubated with Dulbecco’s modified Eagle’s medium HG for 30 mins at 37^o^C. Before cell seeding, plate surfaces were washed twice with sterile PBS. Cells were seeded on a coated surface and divided into two groups, the first group was assigned as normal ECM (N) and cells were maintained in growth medium for 7 days and subsequently maintained in growth medium supplemented with 50 μg/ml L-ascorbic acid for 14 days. The second group was osteogenic ECM group (OM) and cells were maintained in an osteogenic induction medium as described above for 21 days.

After 21 days, cells were decellularized in with 0.5% Triton-X100 in 20 mM ammonium hydroxide for 10 mins and washed twice with sterile PBS and once with sterile deionized water. Subsequently, ECM was incubated with 0.0025% DNase for 30 mins for nucleotide removal.

## Protein extraction and digestion

ECM at day 21 was subjected to protein extraction and isolation using Compartment Protein Extraction Kit (MERCKMillipore, USA). The protein pellets solubilization and digestion were performed essentially as described (Naba et al. 2012) with the following modifications: 13-51 mg of ECM-enriched pellets were solubilized and reduced in a solution of 8M urea, 100 mM ammonium bicarbonate, 10 mM dithiothreitol, pH 8 with vortexing at 37°C for 30 min. After cooling to room temperature, cysteines were alkylated by adding iodoacetamide to 25mM for 30 min. After diluting to 2M urea with 100 mM ammonium bicarbonate pH 8.0, samples were deglycosylated with PNGaseF (New England BioLabs, USA, : 100 units for 1 mg sample) and vortexing at 37°C for 2 hrs, followed by digestion with trypsin/LysC (Promega, USA), at a ratio of 1:10000 enzyme: substrate, with vortexing at 37°C for 2 hrs. Final digestions were done using trypsin (Worthington Biochemical Corporation, USA), at a ratio of 1:1000 (enzyme: substrate), with vortexing at 37°C overnight, followed by a second aliquot of trypsine/LysC (Promega, USA), at a ratio of 1:10000 (enzyme:substrate), and an additional 4 hrs of incubation. Solutions becoming cloudy upon initial reconstitution were cleared after overnight digestion.

## Mass Spectrometry (LC-MS/MS)

Samples were then loaded onto a homemade C18 StageTips for desalting. Peptides were eluted using 40/60 MeCN/H2O + 0.1% formic acid and vacuum concentrated to dryness before LC-MS/MS analysis.

Online chromatography was performed with an RSLCnano system (Ultimate 3000, Thermo Scientific) coupled online to a Q Exactive HF-X with a Nanospay Flex ion source (Thermo Scientific). Peptides were first trapped on a C18 column (75 μm inner diameter × 2 cm; nanoViper Acclaim PepMapTM 100, Thermo Scientific) with buffer A (2/98 MeCN/H_2_O in 0.1% formic acid) at a flow rate of 2.5 μl/min over 4 min. The separation was performed on a 50 cm x 75 μm C18 column (nanoViper Acclaim PepMapTM RSLC, 2 μm, 100Å, Thermo Scientific) regulated to a temperature of 50°C with a linear gradient of 2% to 35% buffer B (100% MeCN in 0.1% formic acid) at a flow rate of 300 nL/min over 211 min. MS full scans were performed in the ultra-high-field Orbitrap mass analyzer in ranges m/z 375–1500 with a resolution of 120,000 at m/z 200. The top 20 intense ions were subjected to Orbitrap for further fragmentation via high energy collision dissociation (HCD) activation and a resolution of 15,000 with the intensity threshold kept at 1 × 105. We selected ions with charge state from 2+ to 6+ for screening. Normalized collision energy (NCE) was set at 27 and the dynamic exclusion of the 40s. For identification, the data were searched against the Homo sapiens (UP000005640) SwissProt database using Sequest HF through proteome discoverer (version 2.2). Enzyme specificity was set to trypsin and a maximum of two-missed cleavage sites were allowed. Oxidized methionine and N-terminal acetylation were set as variable modifications. The maximum allowed mass deviation was set to 10 ppm for monoisotopic precursor ions and 0.02 Da for MS/MS peaks. The resulting files were further processed using myProMS (25) v3.6 (work in progress). FDR calculation used Percolator and was set to 1% at the peptide level for the whole study. The label free quantification was performed by peptide Extracted Ion Chromatograms (XICs) computed with MassChroQ version 2.2 (26). For protein quantification, XICs from proteotypic peptides shared between compared conditions (TopN matching) “and with carbamidomethyl, the modification was used”. Median and scalenormalization was applied on the total signal to correct the XICs for each biological replicate(N=3). To estimate the significance of the change in protein abundance, a linear model (adjusted on peptides and biological replicates) based on a two-tailed T-test was performed and p-values were adjusted using Benjamini–Hochberg FDR procedure. Matrisome proteins (Human Matrisome (Updated August 2014): http://matrisomeproject.mit.edu/other-resources/human-matrisome/) with at least three total peptides in all replicates, a 1,5-fold enrichment and an adjusted p-value < 0.05.

Protein identified and quantified have been deposited to the ProteomeXchange Consortium via the PRIDE (PMID: 30395289) partner repository with the dataset identifier PXD018951.

## Matrisome protein-protein interaction and enrichment pathway analysis

The significant ECM proteins derived from DPSCs detected from matrisome database were analyzed using Metascape -A Gene Annotation & Analysis Resource website- (<https://metascape.org/gp/index.html#/main/step1>). Protein-protein interaction (PPI) enrichment were determined using minimum network size = 3 and maximum network size = 500 with BioGrid+InWeb_IM+OmniPath (human) database. Enrichment of proteins showed by the size of the node, each network is assigned a unique color have been retained as the functional description of pathway and process enrichment analysis. Pathway process following GO term enrichment and canonical pathway analysis followed minimum overlap = 3, *P* value cutoff at 0.01 and minimum enrichment = 1.5. Matrisome proteins were defined as background. Top 10 GO enrichment of common ECM proteins in normal and osteogenic condition were investigated using WEB-based GEne SeT AnaLysis Toolkit.

## Cell viability assay

Cells were incubated with 1 mg/ml of 3-(4,5-Dimethylthiazol-2-yl)-2,5-diphenyltetrazolium bromide (MTT) (USB Corporation) solution for 10 mins at 37°C to allow formazan crystal formation. Further, the formazan crystal was dissolved using dimethyl sulfoxide and glycine buffer. The solution was determined for optical density (OD) by a microplate reader (ELx800; BIO-TEK®) at 570 nm.

## Scanning electron microscopy and energy dispersive X-ray analysis

Ultrastructural and chemical component analyses were performed using SEM (Quanta 250, FEI, Hillsboro, OR, USA). Sample preparation was performed as previously reported (Manokawinchoke et al. 2017). Briefly, specimens were fixed with 3% glutaraldehyde solution for 30 mins and subsequently dehydrated with a graded series of ethanol. Gold sputter-coat were performed for SEM analysis. For those samples evaluated with energy dispersive X-ray (EDX), EDX identified the elemental composition of materials.. The gold coating step was omitted.

## Real-Time Polymerase chain reaction (RT-PCR) Analysis

Total cellular RNA was extracted with Trizol reagent. The quality and quantity of the isolated RNA were evaluated using nanodrop and Qubit. One microgram of RNA sample was converted to cDNA using RT kit (Superscript 2, Life sciences, USA). For real-time quantitative polymerase chain reaction, cDNA was amplified with SYBR green detection system (FastStart Essential DNA Green Master; Roche Diagnostic) on LightCycle®Nano (Roche Diagnostic) or CFX-96 (Bio-Rad). The values of gene expression were normalized to at least two reference gene. Results were analyzed using the Pfaffl method and following the MIQE guidelines. Primer sequences are presented in Appendix Table 1.

## Statistical analysis

Statistical analyses were performed using Prism8 GraphPad software. The data were presented as means ± standard deviations. Statistical significance was assessed by analysis of variance (ANOVA) followed by Turkey’s post hoc test. Differences at p<0.05 were a statistically. Sample from different 3 patients were analyzed by proteomics and 4 different patients were evaluated for the biological properties of decellularized ECM.”

# References

Fournier BP, Loison-Robert LS, Ferre FC, Owen GR, Larjava H, Hakkinen L. 2016. Characterisation of human gingival neural crest-derived stem cells in monolayer and neurosphere cultures. Eur Cell Mater. 31:40-58.

Manokawinchoke J, Nattasit P, Thongngam T, Pavasant P, Tompkins KA, Egusa H, Osathanon T. 2017. Indirect immobilized jagged1 suppresses cell cycle progression and induces odonto/osteogenic differentiation in human dental pulp cells. Sci Rep. 7(1):10124.

Naba A, Clauser KR, Hoersch S, Liu H, Carr SA, Hynes RO. 2012. The matrisome: In silico definition and in vivo characterization by proteomics of normal and tumor extracellular matrices. Mol Cell Proteomics. 11(4):M111 014647.

Osathanon T, Nowwarote N, Pavasant P. 2011. Basic fibroblast growth factor inhibits mineralization but induces neuronal differentiation by human dental pulp stem cells through a fgfr and plcgamma signaling pathway. J Cell Biochem. 112(7):1807-1816.
